# High-density grids for efficient data collection from multiple crystals

**DOI:** 10.1107/S2059798315020847

**Published:** 2016-01-01

**Authors:** Elizabeth L. Baxter, Laura Aguila, Roberto Alonso-Mori, Christopher O. Barnes, Christopher A. Bonagura, Winnie Brehmer, Axel T. Brunger, Guillermo Calero, Tom T. Caradoc-Davies, Ruchira Chatterjee, William F. Degrado, James S. Fraser, Mohamed Ibrahim, Jan Kern, Brian K. Kobilka, Andrew C. Kruse, Karl M. Larsson, Heinrik T. Lemke, Artem Y. Lyubimov, Aashish Manglik, Scott E. McPhillips, Erik Norgren, Siew S. Pang, S. M. Soltis, Jinhu Song, Jessica Thomaston, Yingssu Tsai, William I. Weis, Rahel A. Woldeyes, Vittal Yachandra, Junko Yano, Athina Zouni, Aina E. Cohen

**Affiliations:** a Stanford Synchrotron Radiation Lightsource, SLAC National Accelerator Laboratory, Menlo Park, CA 94025, USA; b Linac Coherent Light Source, SLAC National Accelerator Laboratory, Menlo Park, CA 94025, USA; c Department of Structural Biology, University of Pittsburgh School of Medicine, Pittsburgh, PA 15261, USA; d Art Robbins Instruments, Sunnyvale, CA 94089, USA; e Department of Molecular and Cellular Physiology, Stanford University, Stanford, CA 94305, USA; f Howard Hughes Medical Institute, Stanford University, Stanford, CA 94305, USA; g The ARC Centre of Excellence in Advanced Molecular Imaging, Monash University, Melbourne, Victoria 3800, Australia; h Australian Synchrotron, 800 Blackburn Road, Clayton, Melbourne, Victoria 3168, Australia; i Physical Bioscences Division, Lawrence Berkeley National Laboratory, Berkeley, California, USA; j Department of Pharmaceutical Chemistry, University of California San Francisco, San Francisco, CA 94158, USA; k Department of Bioengineering and Therapeutic Sciences, University of California San Francisco, San Francisco, CA 94158, USA; l Institut für Biologie, Humboldt-Universität zu Berlin, 10099 Berlin, Germany; m Stanford University School of Medicine, Stanford, CA 94305, USA; n Department of Structural Biology, Stanford University, Stanford, CA 94305, USA

**Keywords:** XFELs, high-throughput crystallography, serial crystallography, sample delivery, automation for sample-exchange robots

## Abstract

Higher throughput methods to mount and collect data from multiple small and radiation-sensitive crystals are important to support challenging structural investigations using microfocus synchrotron beamlines. Furthermore, efficient sample-delivery methods are essential to carry out productive femtosecond crystallography experiments at X-ray free-electron laser (XFEL) sources such as the Linac Coherent Light Source (LCLS). To address these needs, a high-density sample grid useful as a scaffold for both crystal growth and diffraction data collection has been developed and utilized for efficient goniometer-based sample delivery at synchrotron and XFEL sources. A single grid contains 75 mounting ports and fits inside an SSRL cassette or uni-puck storage container. The use of grids with an SSRL cassette expands the cassette capacity up to 7200 samples. Grids may also be covered with a polymer film or sleeve for efficient room-temperature data collection from multiple samples. New automated routines have been incorporated into the *Blu-Ice*/*DCSS* experimental control system to support grids, including semi-automated grid alignment, fully automated positioning of grid ports, rastering and automated data collection. Specialized tools have been developed to support crystallization experiments on grids, including a universal adaptor, which allows grids to be filled by commercial liquid-handling robots, as well as incubation chambers, which support vapor-diffusion and lipidic cubic phase crystallization experiments. Experiments in which crystals were loaded into grids or grown on grids using liquid-handling robots and incubation chambers are described. Crystals were screened at LCLS-XPP and SSRL BL12-2 at room temperature and cryogenic temperatures.

## Abstract 2

A high-density sample mount has been developed for efficient goniometer-based sample delivery at synchrotron and XFEL sources.

## 1. Introduction   

As structural biologists tackle ever more challenging systems, the development of efficient methods to deliver large quantities of crystals for X-ray diffraction studies is increasingly important. Proteins that are difficult to crystallize will often produce only small crystals that yield only a few degrees of diffraction data before succumbing to the damaging effects of radiation exposure. For many systems, obtaining a complete data set to high resolution using very small crystals is possible through the use of microfocus synchrotron beams and the collection and combination of data from multiple crystals. For example, when solving the structure of the β_2_ adrenergic receptor–Gs protein complex, hundreds of microcrystals were screened and 20 microcrystals were used in the final data set (Rasmussen *et al.*, 2011 ▸). The structural information accessible from small or radiation-sensitive crystals may be extended through the application of femtosecond crystallography (FX), an emerging method that capitalizes on the extremely bright, short time-scale X-ray pulses produced by X-ray free-electron lasers (XFELs). This approach exploits a ‘diffraction before destruction’ phenomenon (Neutze *et al.*, 2000 ▸) where a still diffraction pattern is produced by a single X-ray pulse before significant radiation-induced electronic and atomic rearrangements occur within the crystal (Barty *et al.*, 2011 ▸; Lomb *et al.*, 2011 ▸). Since the area of the crystal exposed to the X-ray pulse is completely destroyed after each shot, multiple crystals are required for these experiments (Chapman *et al.*, 2011 ▸). In addition, FX provides a means to determine catalytically accurate structures of radiation-sensitive metalloenzymes (Kern *et al.*, 2013 ▸) which may undergo structural rearrangement upon photo-reduction of the metal center at a synchrotron (Peters *et al.*, 1998 ▸; Yano *et al.*, 2005 ▸; Corbett *et al.*, 2007 ▸; Meharenna *et al.*, 2010 ▸; Davis *et al.*, 2013 ▸). In most cases, these experiments also require a large quantity of crystals as each area of the crystal can only be exposed once.

High-throughput crystallization (Yokoyama *et al.*, 2000 ▸; Blundell *et al.*, 2002 ▸; Braun *et al.*, 2002 ▸; Morissette *et al.*, 2004 ▸; Li *et al.*, 2012 ▸) and the implementation of automated sample-mounting systems at synchrotron light sources (Snell *et al.*, 2004 ▸; Cork *et al.*, 2006 ▸; Smith & Cohen, 2008 ▸; Cohen *et al.*, 2014 ▸) have made data collection from multiple crystals approachable; however, challenges still exist. The process of sample exchange using automated mounting systems, which includes mounting a crystal, centering a crystal for data collection and dismounting the crystal, can take between 35 s and a few minutes. While this time scale may be suitable for experiments that require screening of at most a few hundred crystals, higher efficiency methods are critical for more challenging experiments at XFEL sources, where time is limited and in high demand. Furthermore, harvesting crystals for data collection can be another time-consuming step. Crystal-manipulation robots are being developed to automate this process (Heidari Khajepour *et al.*, 2013 ▸; Tung *et al.*, 2014 ▸); however, crystal harvesting is still primarily performed by hand.

Sample injectors allow rapid room-temperature data collection at XFEL sources by delivering a large number of crystals in a liquid jet directly into the path of a series of X-ray pulses (DePonte *et al.*, 2008 ▸; Barty *et al.*, 2011 ▸; Lomb *et al.*, 2011 ▸; Chapman *et al.*, 2011 ▸; Sierra *et al.*, 2012 ▸; Boutet *et al.*, 2012 ▸; Johansson *et al.*, 2012 ▸; Kern *et al.*, 2012 ▸, 2013 ▸, 2014 ▸; Redecke *et al.*, 2013 ▸; Liu *et al.*, 2013 ▸; Barends *et al.*, 2014 ▸). However, most liquid-injector experiments require a substantial number of crystals. Moreover, complications with delivery may arise: the solution may bubble, freeze or dry and disrupt the formation of the stream exiting the injector, and crystals may be damaged by the shear forces of the injection process (Stevenson *et al.*, 2014 ▸). The maximum size of crystals compatible with many injector experiments is limited, and often crystal suspensions must be filtered in advance to remove crystals greater than approximately 5 µm. Also, because the crystals are not cryogenically stored, sample degradation may prevent advance preparation and stockpiling of crystals for the experiment.

An alternative approach for efficient sample delivery and diffraction-quality screening is the use of high-density sample containers that hold crystals in known locations coupled with the use of a high-speed sample goniometer for rapid sample positioning. Examples of high-density sample-mounting containers for room-temperature data collection include microfluidic chips and microcrystal traps (Dhouib *et al.*, 2009 ▸; Kisselman *et al.*, 2011 ▸; Pinker *et al.*, 2013 ▸; Lyubimov *et al.*, 2015 ▸; Roedig *et al.*, 2015 ▸).

Here, we describe a simple, inexpensive high-density sample-mounting grid which enables efficient automated data collection from a large number of crystals that will only survive a few X-ray exposures. The use of these grids to collect a complete, radiation-damage-free data set using myoglobin crystals at the Linear Coherent Light Source (LCLS) XFEL has been described previously (Cohen *et al.*, 2014 ▸). Briefly, hexagonal (*P*6) myoglobin crystals were hand-mounted in grid ports and flash-cooled for data collection at the LCLS X-ray Pump Probe (LCLS-XPP) instrument. To address potential issues with preferential orientation of crystals within grids, data were collected with the grid face oriented at varying angles to the beam. In total, data were collected from 932 crystals in 32 grids, and 739 still images were included in the final data set, which was over 90% complete to 1.36 Å resolution (Cohen *et al.*, 2014 ▸). In this paper, we present an overview of the supporting software routines for grid data collection developed at the Stanford Synchrotron Radiation Lightsource (SSRL) and additional tools that expand the utility of grids to accommodate a wide range of experimental strategies both at cryogenic and room temperature. To illustrate the techniques involved, we detail experiments in which crystals were grown on grids or loaded onto grids using liquid-handling robots for diffraction-quality screening experiments at LCLS-XPP.

## 2. Sample-mounting grids   

**Figure 1:**
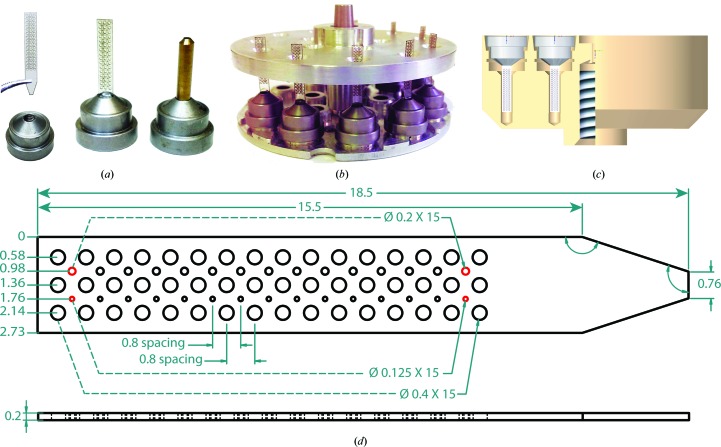
(*a*) The sample-mounting grid is affixed in a standard magnetic base with epoxy. This allows them to be manipulated by automated mounting systems. For comparison, a Hampton Research-style copper magnetic sample pin is also shown. (*b*) A jig is used to hold grids in place as the epoxy cures. The jig consists of two parts: the base piece from a standard uni-puck and a specialized cover machined from aluminium. Guide holes in the cover hold grids vertically as the epoxy sets. (*c*) Cutaway view of a uni-puck enclosure (tan) with two grids (white) inside. (*d*) Schematic of the sample-mounting grid with the fiducial ports highlighted in red. Units are shown in millimetres.

The grid scaffold consists of a piece of 100 or 200 µm thick polycarbonate plastic with laser-cut rows of holes (or ports). This polycarbonate scaffold is affixed to a standard magnetic base to produce the ‘grid assembly’ (Fig. 1 ▸ *a*). A specialized bonding jig is used to hold the polycarbonate scaffold inside the magnetic base as the epoxy sets (Fig. 1 ▸ *b*). Grid ports may hold either large crystals or groups of smaller crystals in known locations. The current grid layout has 75 ports of 400, 200 and 125 µm in diameter (Fig. 1 ▸ *d*); however, the size and arrangement of ports may be altered to better suit different experimental setups. Since crystals are held in known locations, rapid and precise automated crystal positioning into the X-ray beam path is possible.

The outer dimensions of the grid are the largest area that can reliably fit inside the port of an SSRL cassette or uni-puck storage container (Fig. 1 ▸ *c*). The use of grids expands the cassette capacity from 96 to over 7200 sample locations (assuming one crystal per loop/port). The sample-mounting grid enables efficient automated data collection from a large number of crystals that will only survive a few X-ray exposures or small rotational ranges during data collection (Cohen *et al.*, 2014 ▸). Grids may have an additional thin polymer film affixed to one face to better hold samples within ports or to serve as a scaffold for sitting-drop or hanging-drop crystallization experiments (Supplementary Fig. S1). The 5 µm thick polycarbonate film used has minimal X-ray absorption and low X-ray scattering background.

## 3. Crystal loading by hand   

Grids may be manually filled with crystals. To view crystals during this process, it is helpful to position the grid assembly underneath a microscope using a magnetic holder (Supplementary Fig. S2). Grid ports should be prefilled with cryoprotectant oil such as Paratone-N or paraffin to prevent crystal dehydration. A fine needle may be used to apply oil to each grid port. A cryo-loop may be used as a tool to remove a crystal from the crystallization tray, coat the crystal with a thin layer of oil and then transfer it to an appropriately sized grid port. It is helpful to match the size of the cryo-loop tool to a port size in the grid. Filling all ports in a grid may be impractical because crystals may degrade over time in the cryoprotectant oil. Testing is necessary to determine the maximum timeframe for filling grids with a particular sample and oil. This may be accomplished by filling a grid with crystals and recording the loading time for each port. Diffraction data may then be collected and compared for crystals with known exposure times to the oil. Detailed instructions for grid usage may be found at http://smb.slac.stanford.edu/hardware/sample_mounting_grids/.

## 4. Grid adaptor for liquid-handling robots   

**Figure 2:**
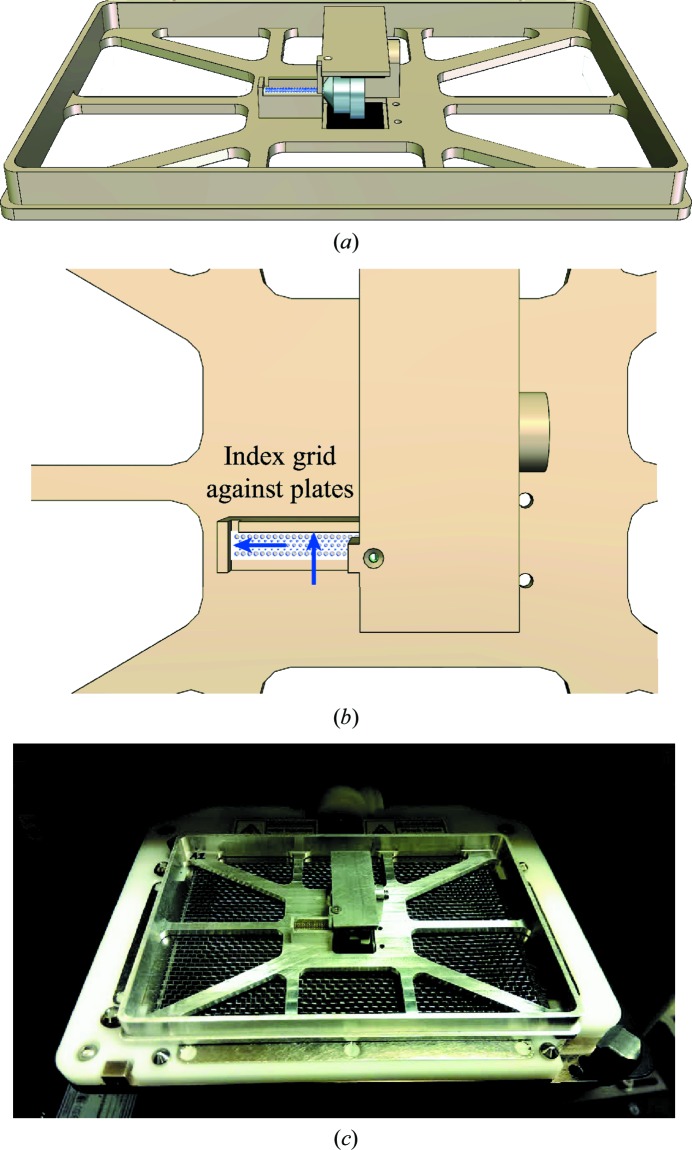
A grid adaptor for liquid-handling robots. (*a*) A grid adaptor with a standard microplate footprint allows grids to be positioned in the plate position of liquid-handling robots, including the ARI Gryphon and the Labcyte Echo 550 liquid handler. A neoprene-lined torsion clip (black) grips the magnetic base and holds the grid in position. (*b*) The grid is indexed against two metal surfaces that protrude from the adaptor to ensure that the ports are correctly positioned. (*c*) A photograph of the grid adaptor holding a grid in the destination-plate position of a Labcyte Echo 550 liquid-handling robot.

The addition of a thin polymer film to one face of the grid creates a scaffold for crystallization experiments. To fill grids with crystallization solutions for this purpose, an adaptor was developed that holds a grid in the destination-plate position of liquid-handling robots (Fig. 2 ▸ *a*). A neoprene-lined torsion clip grips the magnetic base of the grid assembly and holds it in place. Grids are indexed against two metal surfaces to ensure accurate reproducible drop placement (Fig. 2 ▸ *b*). The adaptor has been successfully tested with the Labcyte Echo 550 (Fig. 2 ▸ *c*), the Art Robbins Gryphon (Supplementary Video S1) and the TTP Labtech Mosquito (Supplementary Video S2). After the sample has been deposited on grids, the grids may be incubated in specialized crystal-growth containers which support hanging-drop or sitting-drop experiments and lipidic cubic phase (LCP) crystallization experiments. Liquid-handling robots may also be used to load crystal suspensions onto grids.

## 5. Grid vapor-diffusion chamber   

**Figure 3:**
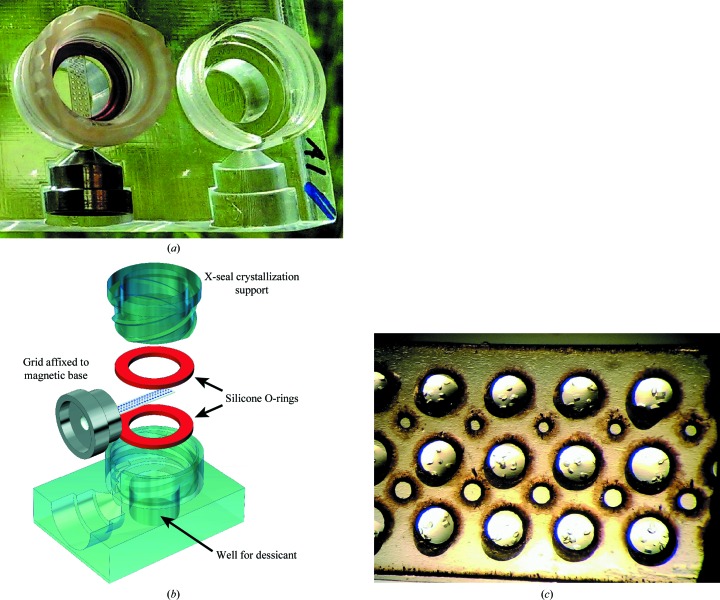
(*a*) A grid incubating inside a vapor-diffusion chamber. Lysozyme crystals are growing in sitting drops on the grid. (*b*) An expanded view of the grid vapor-diffusion chamber. The grid (white) containing sample is held within the crystallization chamber, while the magnetic base (silver) sits in an external cutout. An X-seal crystallization support screws into the crystallization plate to close the chamber. Desiccant may be held below the grid in the chamber. Silicone O-rings (red) create a tight seal around the grid. (*c*) Sitting drops on a grid incubating inside a vapor-diffusion chamber. A thin sheet of polycarbonate was affixed to one face of the grid and a solution of lysozyme in precipitant was dispensed onto the grid backing inline with grid ports. Lysozyme crystals can be seen growing in the sitting drops.

A grid vapor-diffusion chamber was developed to hold a grid in a controlled environment for incubating sitting-drop or hanging-drop crystallization experiments (Fig. 3 ▸ *a*). The chambers are capped with transparent X-seal crystallization supports so that crystal growth may be monitored with a microscope. Silicone O-rings with a thin film of vacuum grease are used to ensure a tight seal around the grid scaffold (Fig. 3 ▸ *b*). A well in the base of the chamber holds up to 350 µl of desiccant below the grid.

## 6. Grid LCP tray   

**Figure 4:**
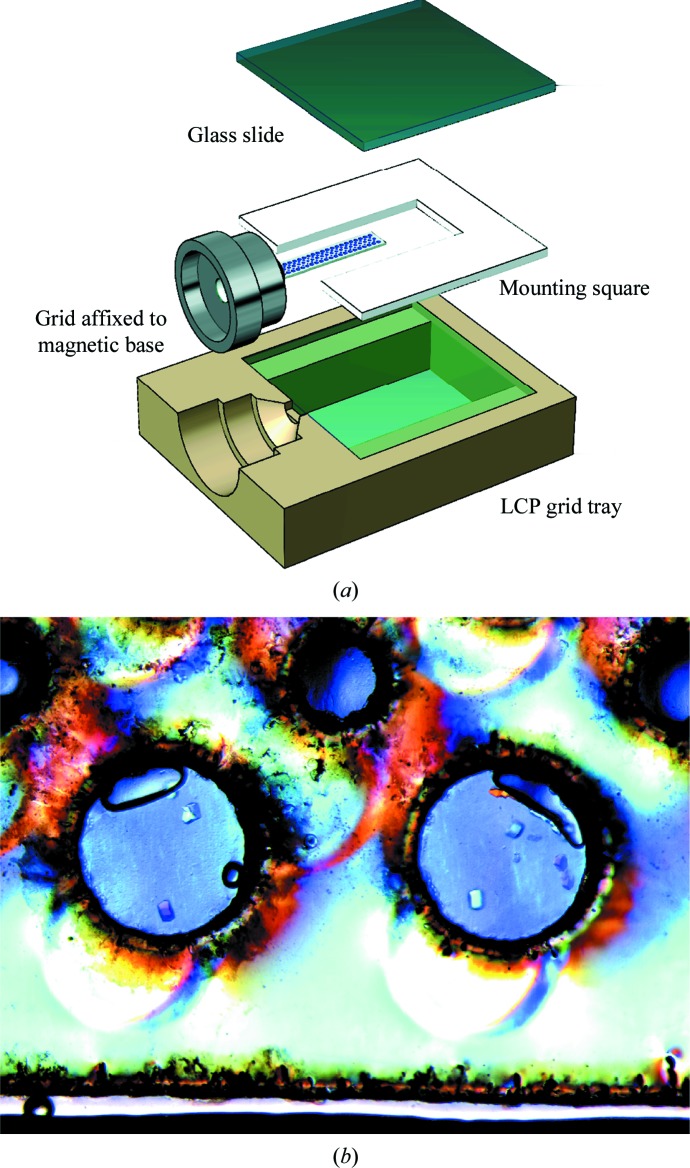
(*a*) Expanded view of the LCP grid tray. The grid (white) containing sample (blue) is sandwiched between two sheets of glass (transparent blue) with precipitating agent surrounding the grid. 1 mm thick double-sided tape (white) with a cutout portion for the grid is used to hold the two sheets of glass together and to create a barrier for the precipitating agent. The assembly sits in a tray (tan) with support cutouts for the glass sandwich and the magnetic base (silver). (*b*) LCP experiments on a grid incubating inside a glass sandwich. Lysozyme crystals can be seen growing in grid ports filled with protein–LCP mixture.

LCP crystallization experiments may also be performed on grids with the use of a specialized tray, eliminating the need to cut open a glass sandwich plate and manually harvest crystals for data collection. The grid LCP tray assembly consists of two siliconized glass slides, 1 mm thick double-sided tape and a tray with a support for a magnetic base and glass slides. The grid is sandwiched between the two sheets of glass and is surrounded by precipitating agent (Fig. 4 ▸ *a*). To aid in removal, a thin polycarbonate sheet may be laid on top of the grid and under the top glass plate.

## 7. Data collection at LCLS   

Grids have been used in combination with goniometer-based instrumentation installed at the LCLS-XPP instrument (Cohen *et al.*, 2014 ▸) for XFEL diffraction experiments. During these experiments, the Stanford Automated Mounter (SAM) robot (Cohen *et al.*, 2002 ▸) was used to mount crystals inside grids onto the goniometer. To control these experiments, automated routines were added to the *Blu-Ice*/*DCSS* experimental control software (McPhillips *et al.*, 2002 ▸; Cohen *et al.*, 2014 ▸). To define the position of all grid ports in relation to the X-ray beam position, a semi-automated alignment procedure takes advantage of the predefined spatial arrangement of the laser-cut grid ports. This process begins by first defining the position of the edge of the grid by rotating it until it is edge-on in the software video display and then clicking on the video image of the grid to move the edge of the grid into the X-ray beam position. If the grid is tilted in this view, two positions may be identified to define the translation path. Next, the grid is rotated by 90° to put the face-on view of the grid in the video image. Four ports on the outer corners of the grid are then clicked in a specified order, which act as fiducial markers to define the port locations and the grid rotation (Supplementary Fig. S3; Fig. 1 ▸ *d*). This procedure calibrates all of the grid ports to the coordinate system of the goniometer and the beam-interaction region.

For crystals that closely match the size of each grid port, data collection may then be carried out automatically; each port is automatically centered into the X-ray interaction region and exposed. Alternatively, automated data collection may be paused and a different area of the port may be selected for exposure using a manual ‘click-and-shoot’ procedure. Helical data collection may also be performed across longer crystals within grid ports (Cohen *et al.*, 2014 ▸). A spreadsheet specifying which grid ports contain crystals may be uploaded in advance so that empty ports are automatically skipped during data collection. ‘Oscillation’ images may be collected by exposing the crystal to attenuated X-ray pulses at 120 Hz while rotating the grid at a constant velocity of 1° s^−1^. An oscillation image collected over 1° is comprised of 120 overlaid still diffraction patterns (Cohen *et al.*, 2014 ▸).

In cases where a group of small crystals is present inside a port, rastering may be performed on the entire grid port or on a smaller area within the port. Rastering is often performed at synchrotrons with low doses of radiation to locate crystals (Song *et al.*, 2007 ▸; Cherezov *et al.*, 2009 ▸). At LCLS, however, rastering is performed with a full dose of radiation to collect data from multiple crystals in a grid port or to collect data from multiple locations on a single crystal. The entire grid port may be rastered automatically by using a pull-down menu to choose the port, and a suitable circular area is automatically defined (Supplementary Fig. S4*a*), or a smaller area containing crystals may be rastered by defining the edges of a polygon from within the software display (Supplementary Fig. S4*b*).

## 8. Data collection at the synchrotron   

Data collection for grids is also supported at the SSRL macromolecular crystallography beamlines, and a tab for grid data collection has been added to the *Blu-Ice*/*DCSS* experimental control software (McPhillips *et al.*, 2002 ▸). Similar to data collection at LCLS, a semi-automated alignment procedure is performed for each grid, after which data collection may proceed. Similar options for automated data collection supported at LCLS also work at SSRL (Supplementary Fig. S3), with the addition of options to collect oscillation data at each crystal position and during X-ray rastering. This automation may be further incorporated into automated workflows (Tsai *et al.*, 2013 ▸) where low-dose X-ray rastering may be used to locate crystals within grid ports for automated multi-crystal data-collection strategies.

Grids are optimal for microcrystal/microbeam data collection at the synchrotron because microcrystals tend to undergo significant radiation damage after only a few degrees of data collection (Rasmussen *et al.*, 2011 ▸). The optimal orientation for the grid is with the face positioned orthogonal to the X-ray beam. When crystals have a preferred orientation within grid ports, a more strategic approach may be taken to achieve completeness by varying the angle of the grid for each exposure. In most cases the grid may be rotated by ±20° or more from the initial position before the resulting diffraction would interact with the polycarbonate scaffold. However, for crystals within the grid ports care must be taken when crystals are positioned at the edge of the port to both avoid interaction of the direct X-ray beam and resulting diffraction with the polycarbonate scaffold. To avoid the latter effect, use of a thin (∼5 µm) polymer sheet affixed to one face of the grid enables the crystallization droplets deposited on the face of the sheeting to be oriented towards the detector so that diffraction occurs away from the grid scaffold (Supplementary Fig. S1).

## 9. Room-temperature data collection with grids   

**Figure 5:**
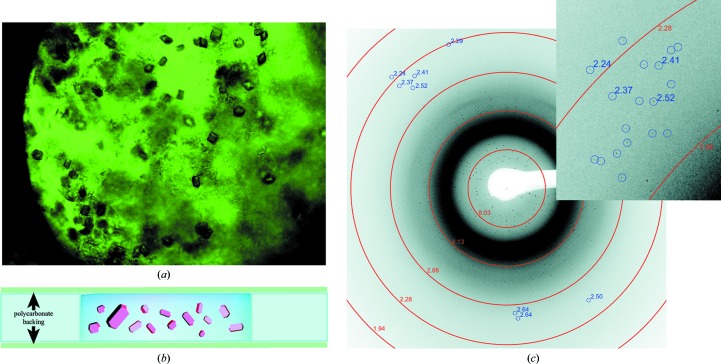
Room-temperature experiments with photosystem II crystals in grids. (*a*) A suspension of photosystem II crystals in a grid port. Polycarbonate backing was applied to one face of the grid, creating a well for a crystal suspension to sit in. A suspension of photosystem II crystals was then pipetted over the open grid ports. (*b*) Schematic of a cross-section of a grid port prepared for a room-temperature experiment. After filling grid ports with crystals, a second sheet of polycarbonate backing may be applied to the grid to seal the grid ports and protect crystals from dehydration. (*c*) Diffraction image of a PSII crystal recorded at LCLS-XPP using the grid mount at room temperature. Resolution is indicated by rings, and individual diffraction spots are highlighted with the corresponding resolution.

Grid ports may be covered with a thin polymer film to prevent evaporation for room-temperature data collection. This approach was used for room-temperature screening of light-sensitive photosystem II (PSII) crystals at LCLS-XPP. Polycarbonate backing was glued to one face of a grid with epoxy. A suspension of PSII crystals was pipetted over the open grid ports, and a loop was used to drag crystals into grid ports (Fig. 5 ▸ *a*). A second sheet of polycarbonate was placed over the open grid ports and held in place by capillary action (Fig. 5 ▸ *b*). Grids were then hand-mounted on the goniometer for data collection at XPP. To avoid exposure of the light-sensitive PSII crystals to ambient light, data collection was performed in the dark. The crystals were substantially smaller (∼20–30 µm) than the grid ports and were difficult to visualize under low light conditions. Therefore, data were collected by rastering the individual 400 µm ports. In a first round of experiments a total of 500 images were collected from three grids. After crystal suspension conditions and data-collection parameters had been optimized, 280 images were collected from a single grid in 18 min and minimal evaporation was observed. Out of this set 87 images contained diffraction data, and the best diffraction was observed to better than 2.5 Å resolution (Fig. 5 ▸ *c*).

## 10. Automated loading of crystal suspensions on grids   

**Figure 6:**
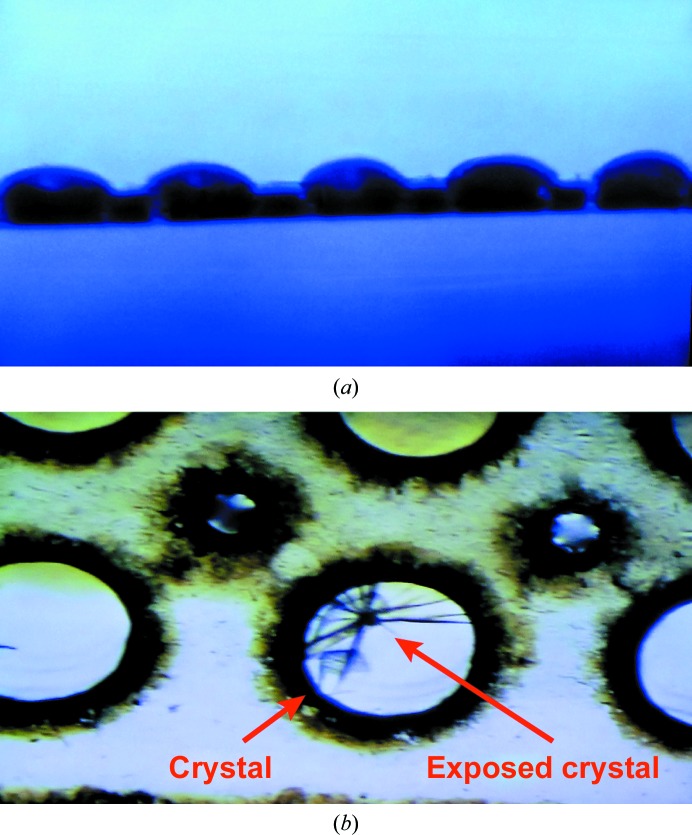
(*a*) An edge-on view of a grid with polycarbonate backing during data collection at LCLS-XPP. An Echo 550 liquid-handling robot was used to position droplets of a perforin crystal suspension inline with grid ports immediately prior to flash-cooling in liquid nitrogen. (*b*) Two crystals of perforin positioned over a grid port during data collection at LCLS-XPP. A hole is clearly visible in the top crystal where it has been exposed to the X-ray beam. The bottom crystal is still intact.

Liquid-handling robots may also be used to dispense suspensions of microcrystals into grids. A suspension of mouse perforin crystals was loaded into grids for screening at LCLS-XPP with the use of an Echo 550 liquid-handling robot. Perforin crystals were prepared as described previously (Law *et al.*, 2010 ▸). The slurry was centrifuged, the supernatant was poured off and the pellet was resuspended in cryprotectant. An Echo 550 liquid-handling robot was used to transfer the suspension onto grids with polycarbonate backing. 30 nl droplets of the crystal suspension were deposited on the surface of the polycarbonate backing inline with each grid port (Fig. 6 ▸ *a*). The grids were immediately flash-cooled and used to screen perforin crystals at LCLS-XPP in December 2014 (Fig. 6 ▸ *b*).

## 11. Vapor-diffusion crystallization experiments on grids   

To test grids as scaffolds for crystal growth, we set up sitting-drop vapor-diffusion experiments on grids with lysozyme. An Echo 550 liquid handler was used to dispense drops of protein and precipitant solution onto a grid with a thin polycarbonate backing. Grids were then incubated in a vapor-diffusion chamber drop-side up with desiccant for 5 d. Lysozyme crystals grew on the grids (Fig. 3 ▸ *c*), and the Echo 550 liquid handler was used to dispense drops of cryoprotectant on top of the crystallization drops.

Vapor-diffusion chambers may also aid in hand-loading crystals that are sensitive to cryoprotectant oils. This approach was recently used to load grids with crystals of RNA polymerase II (Pol II) complexed with general transcription factor IIB (TFIIB) in the presence of a full nucleic acid scaffold bearing 25 noncomplementary bases (TB-25) to simulate a transcription bubble. To accomplish this, a grid was coated with cryoprotectant and placed in a vapor-diffusion chamber with cryoprotectant solution in the well. Two microscopes were used to aid visualization of the crystallization tray and the grid in the vapor-diffusion chamber. Crystals were transferred from the crystallization tray into the grid using cryo-loops. The chamber was loosely sealed between transfers to maintain humidity. The grids were then flash-cooled and used to screen crystals of the Pol II–TFIIB–TB-25 complex both at LCLS-XPP and on SSRL beamline 12-2 (Cohen *et al.*, 2014 ▸).

## 12. Lipidic cubic phase experiments on grids   

Proof of principle LCP crystallization experiments were performed on grids using an adaptation of a protocol for the growth of lysozyme in LCP (Aherne *et al.*, 2012 ▸). An Art Robbins Gryphon was used to dispense cubic phase into grid ports (Supplementary Video S1), and the grids were incubated with precipitating agent in a glass sandwich (Fig. 4 ▸ *b*). Lysozyme crystals up to 50 µm wide were observed in grid ports after 16 h of incubation.

Grids were used to screen for optimal crystal-growth conditions of the influenza M2 transmembrane domain protein at LCLS-XPP. An Art Robbins Gryphon dispensed cubic phase into grid ports, and the volume dispensed into each port was varied across each grid. Grids were then incubated with different precipitating agents in glass sandwiches and crystals were observed growing in the ports (Supplementary Fig. 5 ▸ *a*). The grids were removed from the glass sandwich, covered with polycarbonate sheeting to prevent evaporation and screened at room temperature at LCLS-XPP. A diffraction image is shown in Supplementary Fig. 5 ▸(*b*).

## 13. Discussion   

High-density sample-mounting devices dramatically reduce the amount of time needed for multi-crystal data collection. Data collection of crystals held in loops requires that each crystal be individually mounted on the goniometer, centered in the X-ray beam and then dismounted. Automated alignment of crystal-containing loops can take between 15 and 30 s each, depending on the beamline hardware and software (Sharff, 2003 ▸; Snell *et al.*, 2004 ▸; Pothineni *et al.*, 2006 ▸; Jain & Stojanoff, 2007 ▸; Song *et al.*, 2007 ▸). The time required to mount and dismount a sample varies; however, even if sample exchange requires 25 s, sample exchange, alignment and exposure for 1000 crystals in loops would consume at least 12 h of beam time. Furthermore, 1000 samples mounted individually in loops would require 11 SSRL cassettes or 62 uni-pucks for storage. The sample-mounting grid enables up to 75 conventionally sized crystals (∼100–300 µm in diameter) to be mounted on the goniometer at once, or many thousands of microcrystals, circumventing much of the time involved in sample exchange. Time is further saved because alignment, which takes about 30 s to 1 min depending on the operator, is performed once for the entire grid, after which the position of each sample location is automatically calculated by the *Blu-Ice*/*DCSS* control software (Cohen *et al.*, 2014 ▸). When conventionally sized crystals are used, the use of grids to screen 1000 crystals would reduce the time spent on sample exchange and alignment from about 12 h at best to under 1 h, and a single uni-puck would be sufficient to hold 1000 samples. Additionally, a single grid port may also be filled with multiple microcrystals, in which case thousands of crystals may be mounted on the goniometer at once. In practice, the efficiency of data collection with grids will depend on a number of factors, including the detector readout speed, the number of crystals in each grid, the type of data collection performed and the skill of the user.

Grids are particularly advantageous for room-temperature data collection in which sample exchange is performed manually. In the case of photosystem II, the light sensitivity of the samples imposed additional constraints on the experiment: hand-mounting grids, alignment and data collection were performed in near-darkness, and individual crystals could not be visualized to position them in the beam path. Despite these constraints, 87 diffraction images were obtained from individual 20–30 µm crystals at room temperature in under 1 h including crystal loading, mounting and data collection. In cases where crystals are grown directly on grids, crystal harvesting may be avoided entirely during room-temperature data collection. In the future, the efficiency of room-temperature experiments may be further enhanced through the development of a humidity-controlled sample-storage enclosure that could be developed for the SAM robot in conjunction with a humidity-controlled air stream at the goniometer. Shipment of room-temperature samples to the synchrotron could be achieved with the use of hydrogels in specialized shipping containers to control humidity (Baba *et al.*, 2013 ▸).

A rapidly advancing tool for diffraction experiments is the use of LCP injectors both at XFEL and synchrotron sources (Nogly *et al.*, 2015 ▸). However, thousands of LCP conditions must be screened to find conditions under which well diffracting crystals grow. The use of grids for screening LCP conditions will be a very powerful tool for enabling researchers to use both grids for collection and LCP injectors. The grid is therefore a complementary technique to the LCP injector as it can be used both for direct data collection and for screening LCP conditions.

Further development will focus on fully automating the grid-alignment process using video analysis. To enable automated data collection of crystals held in random locations, low-background fixed-sample mounts and the use of UV tryptophan fluorescence or X-ray rastering techniques to map the positions of randomly positioned crystals against fiducial marks are also under development. The use of these technologies for automated data collection from microcrystals may allow us to match the sample-delivery speed of injectors with negligible sample waste. Furthermore, this automation may be further extended to the development of fully automated workflows for multi-crystal experiments where the data collected automatically using multiple crystals are analyzed for completeness in real time.

## 14. Conclusions   

The use of high-density containers such as grids that hold crystals in known locations enables efficient highly automated data-collection strategies. Adaptors and specialized grid holders allow crystallization experiments to be performed inside grids, bypassing the tedious step of harvesting and mounting crystals. This approach is particularly attractive for very small and delicate crystals.

## 15. Related literature   

The following references are cited in the Supporting Information for this article: Caffrey & Cherezov (2009 ▸), Hellmich *et al.* (2014 ▸) and Pullara *et al.* (2013 ▸).

## Supplementary Material

Supporting Information.. DOI: 10.1107/S2059798315020847/yt5086sup1.pdf

Supplementary Video S1. An Art Robbins Gryphon liquid-handing robot dispensing LCP into grid ports. A grid adaptor with a standard microplate footprint holds the grid in the destination-plate position of the Gryphon.. DOI: 10.1107/S2059798315020847/yt5086sup2.mov

Supplementary Video S2. A TTP Labtech Mosquito liquid-handing robot filling grid ports with LCP. A grid adaptor with a standard microplate footprint holds the grid in the destination-plate position of the Mosquito. (Video credit: Joby Jenkins, TTP Labtech.). DOI: 10.1107/S2059798315020847/yt5086sup3.mov
