# Supplementary material for: High-density grids for efficient data collection from multiple crystals
Source: Acta Crystallogr D Struct Biol. 2016 Jan 1;72(Pt 1):2–11. doi: 10.1107/S2059798315020847 (PMC4756618; doi:10.1107/S2059798315020847)
Supplement: Supplementary file 1 [file d-72-00002-sup1.pdf]

STRUCTURAL BIOLOGY

Volume 72 (2016)

Supporting information for article:

High-density grids for efficient data collection from multiple crystals

Elizabeth L. Baxter, Laura Aguila, Roberto Alonso-Mori, Christopher O. Barnes, Christopher A. Bonagura, Winnie Brehmer, Axel T. Brunger, Guillermo Calero, Tom T. Caradoc-Davies, Ruchira Chatterjee, William F. Degrado, James S. Fraser, Mohamed Ibrahim, Jan Kern, Brian K. Kobilka, Andrew C. Kruse, Karl M. Larsson, Heinrik T. Lemke, Artem Y. Lyubimov, Aashish Manglik, Scott E. McPhillips, Erik Norgren, Siew S. Pang, S. M. Soltis, Jinhu Song, Jessica Thomaston, Yingssu Tsai, William I. Weis, Rahel A. Woldeyes, Vittal Yachandra, Junko Yano, Athina Zouni and Aina E. Cohen

S1. Myoglobin

Crystallization and data collection of myoglobin was performed as described previously (Cohen et al., 2014). Myoglobin was purchased from Sigma.

S2. Photosystem II

Crystals of photosystem II were grown as described previously (Hellmich et al., 2014) and were transferred to a final buffer of 35% PEG5000, 0.1 M NH_3SO_4, 0.1M TRIS. Grids were prepared by covering one face with a polycarbonate backing, and crystals were then pipetted into the 400 µm holes of the grid. Immediately after pipetting the crystal suspension into the grid, the open side was covered with a polycarponate film to prevent dehydration. Grids were hand mounted onto the LCLS-XPP goniometer (Cohen et al., 2014). Data were collected at room temperature by rastering grid ports using 9.5 keV, 2.6 mJ, <50 fs X-ray pulses with a beam size of 10 µm and a step size of 50 µm.

S3. Mouse Perforin

Mouse perforin crystals were grown as described previously (Law et al., 2010) by seeding sitting drops. Crystals were harvested from the drops and then resuspended in a cryo-protectant solution consisting of 30% glycerol, 0.5M Sodium Acetate, 0.1M imidazole, pH 7.0. A 5 µm thick polycarbonate backing was affixed to grids by partially dissolving the surface of the backing with acetone and pressing a grid on to the wet surface. An Echo 550 liquid handling robot was used to dispense 25 nL drops of the crystal suspension onto the grids immediately prior to flash cooling in liquid nitrogen.

S4. Vapor Diffusion Experiments with Lysozyme

5μm thick polycarbonate backing was affixed to grids by partially dissolving the surface of the grid with acetone and pressing the wet face of the grid onto the backing. An Echo550 liquid handler was used to dispense 25nL drops of 30mg/mL lysozyme in 500mM NaCl and 25mM Sodium Acetate pH 4.5 onto the backing over each grid port. Grids containing sample were then incubated in a grid vapor diffusion chamber, (Figure 3A), with 300μL well solution of 0.8M NaCl, 50mM Sodium Acetate pH 4.5. The resulting lysozyme crystals are shown in Figure 3C. After 5 days of incubation, the Echo550 was used to dispense 10nL drops of cryoprotectant solution of 50% v/v ethylene glycol and well solution on to grid ports containing lysozyme crystals. Grids were then immediately flash frozen.

S5. Pol II-TFIIB-TB-25

Pol II and TFIIB were purified and assembled with TB-25 (Pullara et al., 2013) and crystals were grown by seeding sitting drops as described previously. (Stevenson et al., 2014). Crystals were incubated overnight in a cryo-protectant solution consisting of 55% Tacsimate and 5% glycerol, and then washed in a solution of 60% Tacsimate, 5% glycerol. Grids were prepared to receive crystals by coating them with a solution of 60% tacsimate, 5% glycerol. Coated grids were then placed in the vapor diffusion chamber (figure 4A,B) with 60% tacsimate, 5% glycerol in the well, and the well was loosely sealed with an X-seal crystal cap to prevent evaporation. Two microscopes were used one to visualize the crystallization tray and one to visualize the grid. Crystals were then transferred from the crystallization tray into the grid using Hampton cryo-loops. The chamber was loosely sealed between transfers to maintain humidity. Once filled, grids were flash frozen and

stored for data collection. Data were collected as described previously (Cohen et al., 2014).

S6. LCP experiments with Lysozyme

Lysozyme was purchased from Sigma. A modified version of a protocol for growing lysozyme crystals in LCP was performed (Aherne et al., 2012). Grids with a 6μm polycarbonate backing were positioned in the grid adaptor (Figure 2B), and 400nL of cubic phase was dispensed into grid ports using an Art Robbins Gryphon (Supplemental Video 1). The LCP tray was assembled and precipitant was pipetted in to the glass sandwich. The grid was then inserted into the assembly and the edges were sealed with vacuum grease. LCP crystals up to 50 μm wide were observed in grid ports after 16 hours of incubation (Figure 4B).

S7. LCP experiments with Influenza A M2 protein transmembrane domain peptide

Influenza A M2 protein transmembrane domain peptide (residues 22-46) was manually synthesized using Fmoc chemistry. The resulting peptide was reconstituted into the lipidic cubic phase with some modification to the protocol used by Caffrey and Cherezov (Caffrey & Cherezov, 2009). Synthetic peptide was mixed with monoolein in ethanol, then the excess ethanol was removed by lyophilization and the lipid/peptide mixture was mixed with aqueous solution at a ratio of 20 uL solution to 30 mg lipid/peptide at 40 ˚C to make the lipidic cubic phase. M2 in LCP was dispensed onto grids in 100 nL drops using a TTP Labtech LCP Mosquito robot. Precipitant solution was added manually with a pipette at a ratio of 1 µL precipitant per 100 nL drop. The precipitant solution used was 0.2 M CaCl2, 0.1 M Tris pH 8.0, 44% PEG 400. The grids were incubated in a vapor diffusion chamber and 5-10 micron crystals were observed after 1 week.

References

Aherne, M., Lyons, J. A., & Caffrey, M. (2012). *J. Appl. Crystallogr.* **45**, 1330–1333.

Caffrey, M. & Cherezov, V. (2009). *Nat. Protoc.* **4**, 706–731.

Cohen, A. E., Soltis, S. M., González, A., Aguila, L., Alonso-Mori, R., Barnes, C. O., Baxter, E. L., Brehmer, W., Brewster, A. S., Brunger, A. T., et al. (2014). *Proc. Natl. Acad. Sci. U. S. A.* **111**, 17122–17127.

Hellmich, J., Bommer, M., Burkhardt, A., Ibrahim, M., Kern, J., Meents, A., Müh, F., Dobbek, H., & Zouni, A. (2014). *Structure*. **22**, 1607–1615.

Law, R. H. P., Lukoyanova, N., Voskoboinik, I., Caradoc-Davies, T. T., Baran, K., Dunstone, M. A., D'Angelo, M. E., Orlova, E. V, Coulibaly, F., Verschoor, S., et al. (2010). *Nature*. **468**, 447–451.

Pullara, F., Guerrero-Santoro, J., Calero, M., Zhang, Q., Peng, Y., Spåhr, H., Kornberg, G. L., Cusimano, A., Stevenson, H. P., Santamaria-Suarez, H., et al. (2013). *Protein Expr. Purif.* **87**, 111–119.

Stevenson, H. P., Makhov, A. M., Calero, M., Edwards, A. L., Zeldin, O. B., Mathews, I. I., Lin, G., Barnes, C. O., Santamaria, H., Ross, T. M., et al. (2014). *Proc. Natl. Acad. Sci. U. S. A.* **111**, 8470–8475.

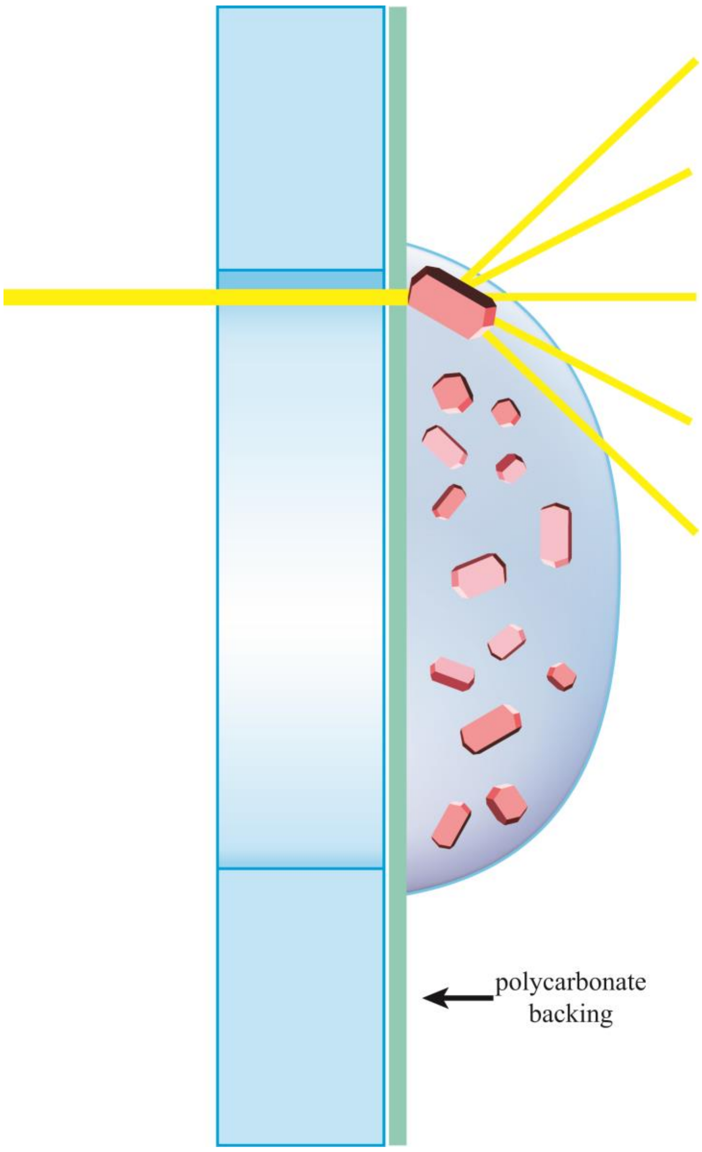

Figure S1 Grid Orientation and Sample Positioning. Pictured is a cross section of a grid port with polycarbonate backing applied to one face of the grid. Sitting drop experiments may be set up on the surface of the polycarbonate backing. Suspensions of crystals may also be deposited on the surface of the polycarbonate backing with the use of liquid handling robots.

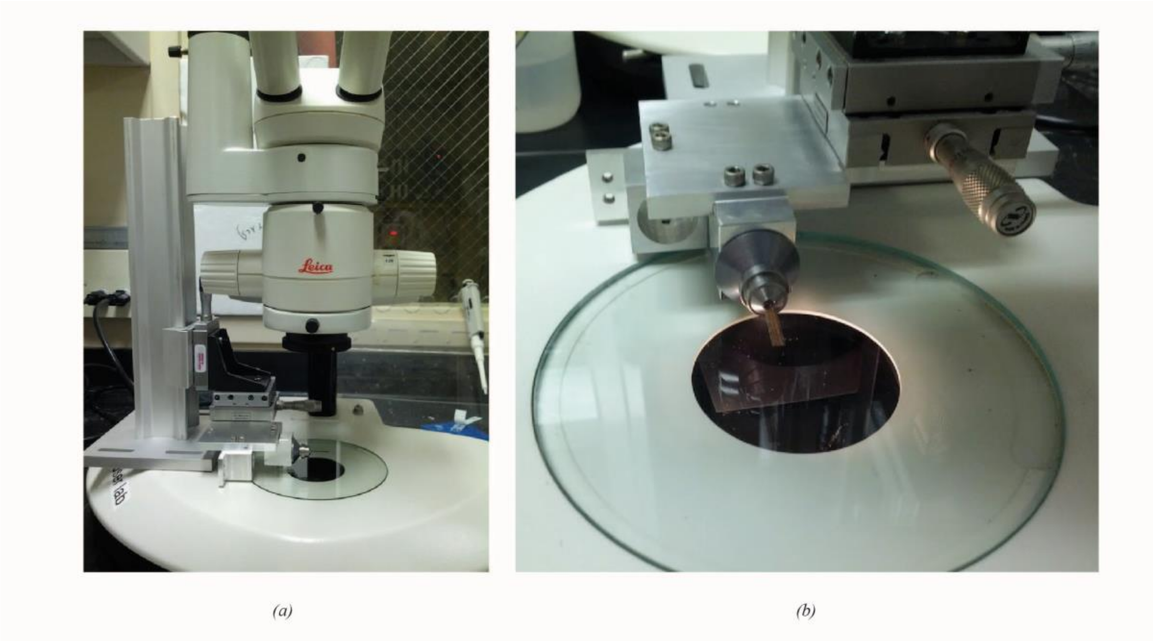

Figure S2 A grid is positioned underneath a microscope with the aid of a magnetic holder.

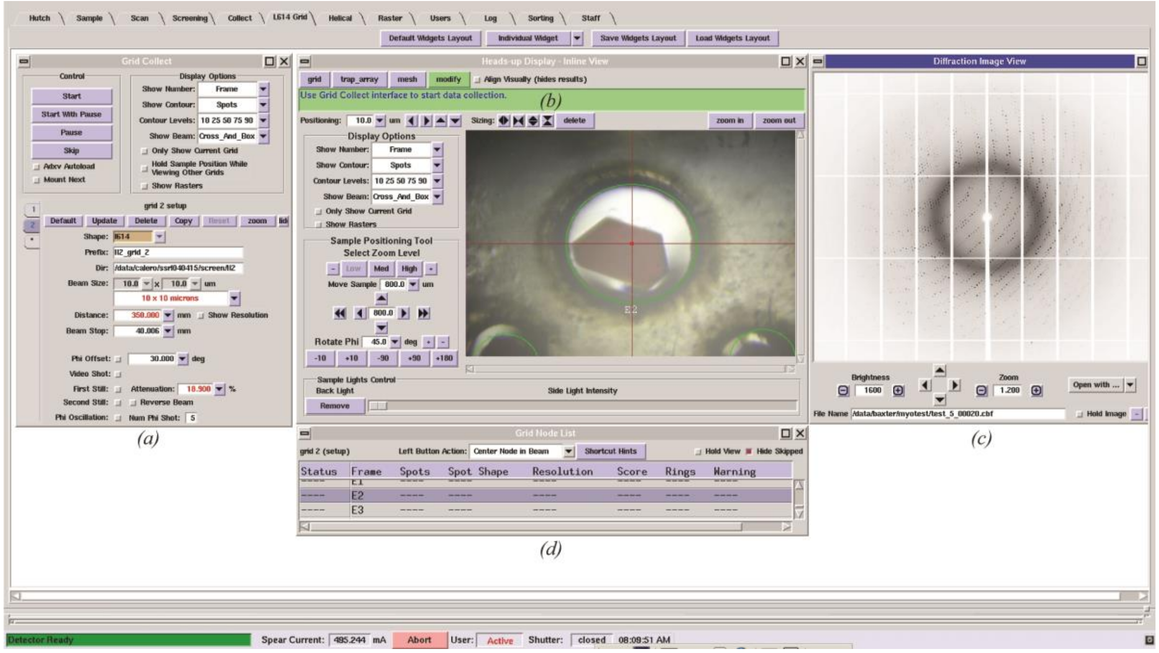

Figure S3 Screen capture of the ‘Grid’ data collection tab in Blu-Ice. **(a)** Collection parameters are specified in the Grid Collect widget. **(b)** Interface for sample positioning and performing semi-automated grid alignment. After alignment is performed, green circles are overlaid onto the video display to indicate the calibrated positions of grid ports. **(c)** Ports may be selected from a

list and automatically centered into the X-ray beam in the Grid Node Widget. The drop down menu also includes an option to automatically draw a circular rastering area around a selected grid port. **(d)** Diffraction Image Viewer

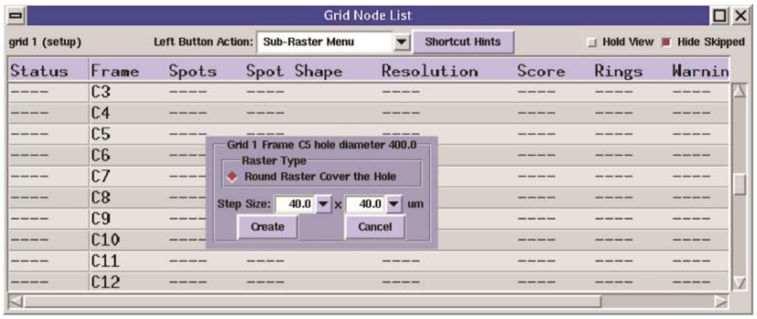
Grid Node List
grid 1 (setup)
Left Button Action:
Sub-Raster Menu
Shortcut Hints
Hold View
Hide Skipped
Status
Frame
Spots
Spot Shape
Resolution
Score
Rings
Warnin
C3
C4
C5
C6
C7
C8
C9
C10
C11
C12
Grid 1 Frame C5 hole diameter 400.0
Raster Type
Round Raster Cover the Hole
Step Size:
40.0
x
40.0
um
Create
Cancel

(a)

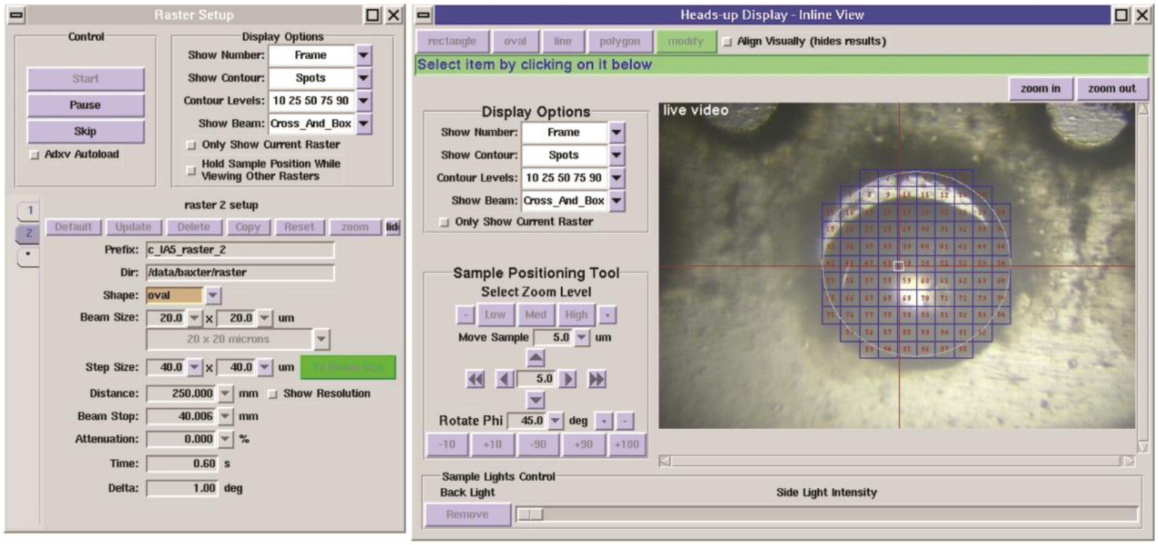
Raster Setup
Control
Start
Pause
Skip
Adxv Autoload
Display Options
Show Number:
Frame
Show Contour:
Spots
Contour Levels:
10 25 50 75 90
Show Beam:
Cross_And_Box
Only Show Current Raster
Hold Sample Position While Viewing Other Rasters
raster 2 setup
Default
Update
Delete
Copy
Reset
zoom
Prefix:
c_IA5_raster_2
Dir:
/data/baxter/raster
Shape:
oval
Beam Size:
20.0
20 x 20 microns
Step Size:
40.0
Distance:
250.000
mm
Show Resolution
Beam Stop:
40.006
Attenuation:
0.000
%
Time:
0.60
s
Delta:
1.00
deg
Heads-up Display - Inline View
rectangle
oval
line
polygon
modify
Align Visually (hides results)
Select item by clicking on it below
zoom in
zoom out
live video
Sample Positioning Tool
Select Zoom Level
Low
Med
High
Move Sample
5.0
um
Rotate Phi
45.0
-10
+10
-90
+90
+180
Sample Lights Control
Back Light
Side Light Intensity
Remove

(b)

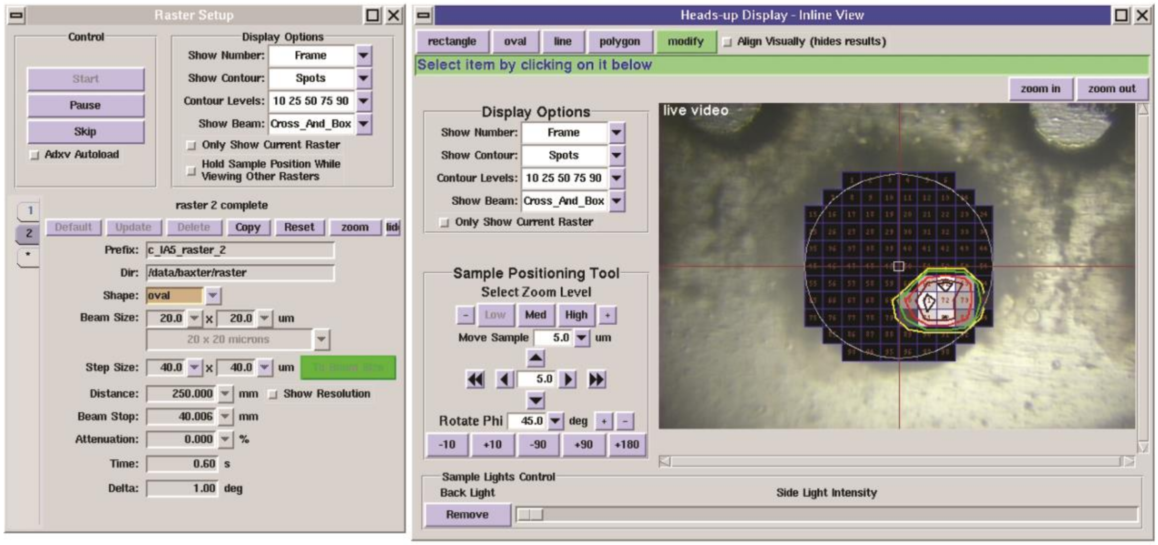
Raster Setup
Control
Start
Pause
Skip
Adxv Autoload
Display Options
Show Number:
Frame
Show Contour:
Spots
Contour Levels:
10 25 50 75 90
Show Beam:
Cross_And_Box
Only Show Current Raster
Hold Sample Position While Viewing Other Rasters
raster 2 complete
Default
Update
Delete
Copy
Reset
zoom
Prefix:
c_IA5_raster_2
Dir:
/data/baxter/raster
Shape:
oval
Beam Size:
20.0
20 x 20 microns
Step Size:
40.0
Distance:
250.000
mm
Show Resolution
Beam Stop:
40.006
Attenuation:
0.000
%
Time:
0.60
s
Delta:
1.00
deg
Heads-up Display - Inline View
rectangle
oval
line
polygon
modify
Align Visually (hides results)
Select item by clicking on it below
zoom in
zoom out
live video
Sample Positioning Tool
Select Zoom Level
Low
Med
High
Move Sample
5.0
um
Rotate Phi
45.0
-10
+10
-90
+90
+180
Sample Lights Control
Back Light
Side Light Intensity
Remove

(c)

Figure S4 Screen captures of the Blu-Ice GUI illustrating the rastering process. **(a)** A rastering area may be automatically drawn around a grid port selected in the Grid Node Widget by selecting the "sub-raster menu" option from the drop down menu. **(b)** A rastering area is drawn over a sitting drop experiment containing lysozyme crystals on a grid for data collection at beamline 12-2. **(c)** Diffracting regions of the rastering area are highlighted and overlaid onto the visual display. Contour lines are added to indicate regions of higher diffraction quality.

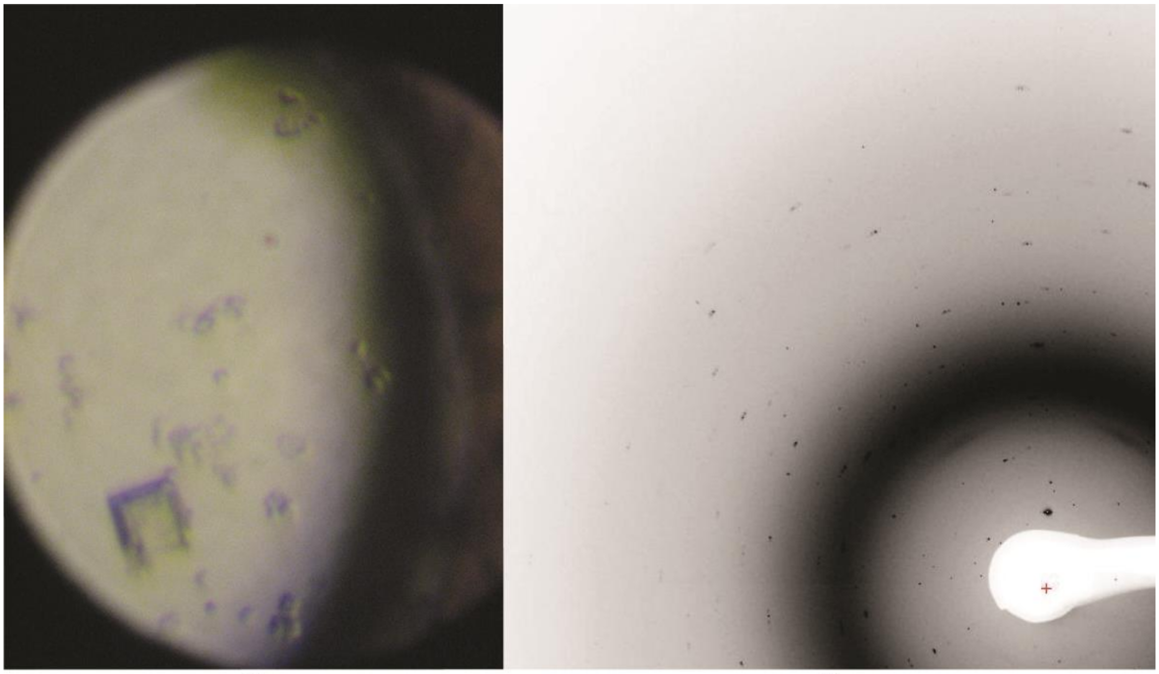

(a) *(b)*

Figure S5 **(a)** LCP experiments with M2 transmembrane domain protein on a grid incubating inside of a glass sandwich. Crystals can be seen growing in grid ports filled with protein-laden mesophase. **(b)** A diffraction image collected during Protein Crystal Screening (PCS) time at LCLS-XPP in December 2014 from an M2 transmembrane domain crystal grown inside a grid.
